# Breast cancer prevention in patients with *gBRCA*-mutated ovarian cancer

**DOI:** 10.1007/s00432-026-06543-4

**Published:** 2026-06-21

**Authors:** Niklas Amann, Annika Krückel, Lena Brückner, Julia Gocke, Carolin Müller, Felix Heindl, Carolin C. Hack, Katharina Stumpf, Julius Emons, Peter A. Fasching, Matthias W. Beckmann, Manuel Hörner

**Affiliations:** 1 Department of Gynecology and Obstetrics, Universitätsklinikum Erlangen, Comprehensive Cancer Center Erlangen-EMN (CCC ER-EMN), Friedrich-Alexander-Universität Erlangen-Nürnberg (FAU), Erlangen, Germany; 2 Bavarian Cancer Research Center (BZKF), Universitätsstraße 21/23, 91054 Erlangen, Germany

**Keywords:** BRCA, Hereditary breast and ovarian cancer, Intensified breast cancer surveillance, Metachronous breast cancer, Risk-reducing bilateral mastectomy, Ovarian cancer

## Abstract

**Purpose:**

Ovarian cancer is frequently associated with *gBRCA1/2* mutations, which also confer a high lifetime risk of breast cancer in non-affected patients. While risk-reducing salpingo-oophorectomy (RRSO) is established in hereditary breast and ovarian cancer prevention, the recommendation for risk-reducing bilateral mastectomy (RRBM) remains unclear in *gBRCA1/2*mutovarian cancer patients due to high recurrence rates and limited survival. This study evaluates the preventive strategies of these patients in a real-world setting.

**Methods:**

This retrospective study included 49 patients with *gBRCA1/2mut* HGSC treated at the Hereditary Breast and Ovarian Cancer Centre, University Hospital Erlangen, between 2012 and 2024. Clinical, pathological, treatment-related, and genetic data were collected and analyzed from electronic records.

**Results:**

Among 49 patients with *gBRCAmut* HGSC, 44 out of 49 did not undergo a RRBM during follow-up. Intensified breast cancer surveillance was recommended to 33 patients. Although 19 out of 49 patients were formally recommended a RRBM, only two proceeded with the preventive option. Three additional patients chose the surgery driven by the diagnosis of metachronous breast cancer. 80% of the operations occurred > 60 months after ovarian cancer diagnosis.

**Conclusions:**

In patients with *gBRCA1/2mut* and a history of HGSC, RRBM is rarely chosen. Most patients prefer an intensified surveillance program. Due to low metachronous breast cancer rate in follow up care, RRBM seems to be an option just for long-term survivors. Individual patient preferences play a crucial role in the management strategy.

**Supplementary Information:**

The online version contains supplementary material available at https://doi.org/10.1007/s00432-026-06543-4.

## Introduction

Globally, more than 250,000 women are diagnosed annually with high-grade serous carcinoma of the ovary, peritoneum or fallopian tube (HGSC) (Reid et al. 2017). In Germany, approximately 7,000 new cases are reported each year. With more than 200,000 deaths per year, this cancer represents the second most common cause of death among gynaecologic malignancies worldwide, following cervical cancer (Sung et al. 2021).

In approximately 50% of HGSC cases, a deficiency in the homologous recombination DNA repair pathway is considered a key pathogenic mechanism (Radhakrishnan et al. 2014). Germline and somatic mutations in the *BRCA1* and *BRCA2* genes, which are part of this pathway, can be identified in approximately 20% of all HGSCs (Kanchi et al. 2014, Ataseven et al. 2020). Carriers of pathogenic *gBRCA* mutations have not only an increased risk for HGSC but also a markedly elevated lifetime risk for breast cancer (BC). The estimated cumulative BC risk is approximately 70% for *gBRCA1* and *gBRCA2* mutation carriers (Kuchenbaecker et al. 2017). Overall, approximately 10% of all BC cases are attributed to hereditary breast and ovarian cancer (HBOC) syndrome (Ataseven et al. 2020, Beckmann et al. 1997).

Risk-reducing surgical interventions can substantially lower the incidence of BC and ovarian cancer (OC). Additionally, they reduce disease-specific mortality and improve overall survival (Metcalfe et al. 2024, Domchek et al. 2010, Rebbeck et al. 2004, Meijers-Heijboer et al. 2001). Risk-reducing bilateral mastectomy (RRBM) is recommended for breast cancer prevention in *gBRCA*mut patients, risk-reducing bilateral salpingo-oophorectomy (RRSO) for ovarian cancer prevention. However, in patients already been diagnosed with HGSC, there is an uncertainty whether a subsequent RRBMshould be recommended or not. The high risk of HGSC recurrence within the first 18 to 24 months after diagnosis often precludes clear recommendations on prophylactic breast surgery (Perren et al. 2011, Burger et al. 2011, González-Martín et al. 2023, Caruso et al. 2025, Salas Bolívar et al. 2025).

More recent evidence suggests the incidence of breast cancer in OC follow-up is exceedingly low (Apostol et al. 2026). The BC related deaths in *gBRCA*mut HGSC seems to be less than 1% (Apostol et al. 2026). Accordingly, Apostol et al. propose that RRBM should only be considered in long-term survivors. As a more favourable alternative, intensified breast cancer surveillance (IBCS) is recommended for these patients.

The primary objective is the description of real-world data on BC preventive strategies in patients with *gBRCA*mut HGSC, the inclusion in IBCS and surgical interventions. As secondary objectives metachronous BC cases during follow-up and surgical interventions (indication, time to operate, operation procedure) are analyzed. New scientific evidence should be substantiated with data derived from routine clinical practice.

## Methods

### Study site

Between January 1, 2012, and December 31, 2024, 3,599 individuals presented at the HBOC Centre at the University Hospital Erlangen. The number of patients increased continuously over the study period. The official certification of HBOC center was associated with a further significant increase in patient enrollment between 2021 and 2023. Consequently, 629 genetic tests were conducted in 2023 (Amann et al. 2025). In order to qualify for genetic counseling related to HBOC, patients had to fulfill the testing criteria established by the German Cancer Society. A distinction is made between affected and unaffected individuals, with preventive, diagnostic, or therapeutic indications justifying germline testing.

Patients diagnosed with breast or ovarian cancer must also meet the German Cancer Society’s testing criteria. These criteria say genetic testing is recommended if breast cancer was diagnosed before the age of 36 years, if triple-negative breast cancer was diagnosed before the prespecified cut-off age, which changed over the years, or if ovarian/peritoneal/fallopian tube cancer was diagnosed before the age of 80 years, even in the absence of a suspicious family history. Genetic testing should furthermore be offered to individuals from families with three cases of BC, or with two cases of BC if at least one diagnosis was made before the age of 50 years. While these criteria are broadly consistent with international guidelines, they do not fully align with the recommendations of the National Comprehensive Cancer Network or the American Society of Clinical Oncology. Consistent with the recommendations of the German Cancer Society, genetic testing is indicated if the probability of identifying a pathogenic germline variant is at least 10%. These criteria are derived from the work of Kast et al. (Kast et al. 2016).These criteria are reviewed annually and adapted based on the latest scientific evidence, resulting in changes to the testing guidelines during the study period. Following the introduction of poly ADP ribose polymerase inhibitors (PARPi) for the locally advanced ovarian cancer and human epidermal growth factor receptor 2-negative breast cancer associated with pathogenic *BRCA1/2* mutations, germline testing also gained therapeutic relevance irrespective of family history.

### Patients

**Fig. 1:**
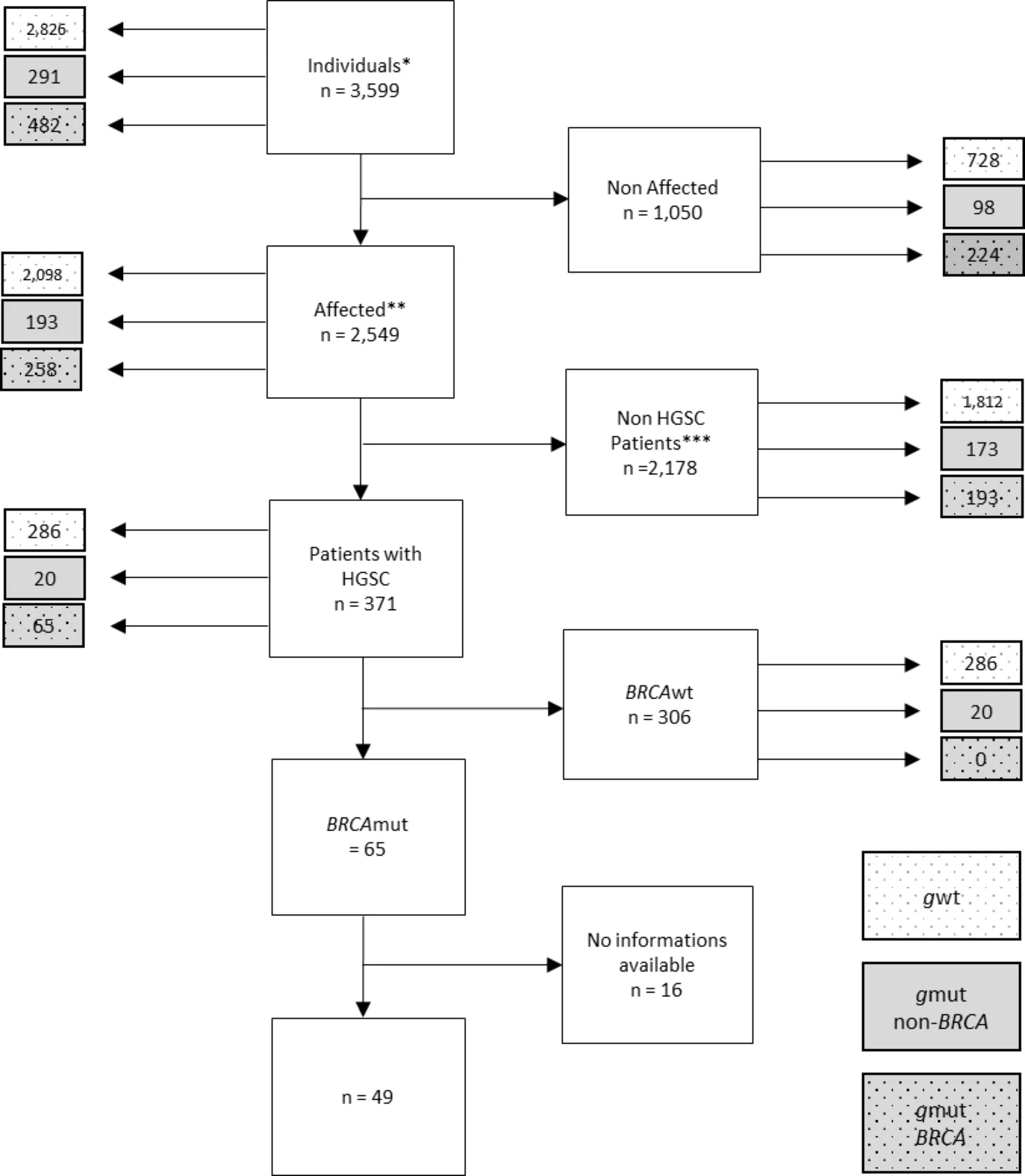
Patient selection for *gBRCAmut* HGSC out of 3,599 patients tested for hereditary breast and ovarian cancer at University Hospital Erlangen. * = positive HBOC criteria (preventive, diagnostic, therapeutic testing), ** = Diagnosed Cancer, *** = Borderline-Tumours, Breast Cancer, Ductal Carcinoma in situ

At the time of data cut-off (December 31, 2024), 3,599 patients were registered in the database. Of these, non-affected individuals (*n* = 1,050), affected patients with non-ovarian cancer (*n* = 2,178), and patients without a *gBRCA* mutation (*n* = 306) were excluded. A *gBRCA* mutation was detected in 65 patients with HGSC. From this population, data were unavailable for an additional 16 patients. These 16 patients were just tested for HBOC but nether been treated for OC at University Hospital Erlangen. Ultimately, 49 patients with *gBRCAmut* HGSC were included in this analysis. Epidemiological, clinicopathological, and treatment-related data regarding the cancer diagnosis and treatment were assessed. Furthermore, information on germline mutation analysis and subsequent management—specifically participation in IBCS programmes or the performance of RRBM—was collected and evaluated. RRBM was evidenced-based recommended due to national guideline depending on the age the patients. The patient flow chart is shown in Fig. 1.

### Structured process for genetic testing

Patients eligible for genetic counseling are identified through a systematic assessment of the German Cancer Society’s inclusion criteria. If the criteria are met, patients can undergo counseling at the HBOC centre. The counseling is provided by trained medical personnel who have completed the GCHBOC basic module and hold additional qualifications in specialized human genetics counseling.

Upon completion of the counseling session and after obtaining written informed consent, genetic testing is initiated. A blood sample is collected and analyzed using next-generation sequencing technology. The specific methods employed are detailed in the molecular genetic report, which also includes information about the genes analyzed and any identified genetic alterations. More than one genetic variant is possible. Genetic variants are classified according to the current Human Genome Variation Society nomenclature.

Once the molecular genetic results are available, findings are discussed with the patient. In cases where a pathogenic or likely pathogenic variant is detected (classified as ACMG class 4 or 5), risk-reducing surgical options (e.g., mastectomy, adnexectomy) or enrollment in an IBCS programme are considered, depending on the specific gene mutation. If the variant is of uncertain significance (ACMG class 3), patients are advised to return for re-evaluation of the variant after three years. No immediate therapeutic or diagnostic measures are taken in such cases. A negative (non-pathogenic) result has no clinical implications. It should be emphasized that certain patient groups who meet the criteria for genetic testing may remain eligible for additional interventions even if their test results are negative or of uncertain significance. For instance, in Germany, patients with breast cancer diagnosed before the age of 45 years may still participate in the IBCS programme until they reach the age of 50 years, irrespective of genetic findings.

### Data collection and statistical analysis

Patient data from the period between January 1, 2012, and December 31, 2024, at the HBOC Centre at the University Hospital Erlangen, were collected retrospectively. Medical histories and results of human genetic testing were documented in an electronic case report form using Microsoft Access 365 (Microsoft, Redmond, WA, USA). Patient and tumour characteristics were described using summary statistics. Frequency and percentage were used for categorical variables.

## Results

### Patient characteristics

The final study cohort comprised 49 patients with *gBRCAmut* HGSC. In 34 patients (69.3%) a *BRCA1* mutation was detected, in 15 patients a *BRCA2* mutation (Supplementary Table 1 shows the coding and protein change). Demographic and baseline tumour characteristics are shown in Table 1. The mean age at diagnosis was 58.7 years. A total of 71.4% of patients were diagnosed aged 50–70 years. Eight patients were aged < 50 years at the time of diagnosis, and 6 patients were aged > 70 years. Approximately one-third of the patients were additionally diagnosed with metachronous BC, four patients during the course of follow-up. By December 2024, 40.9% of patients (*n* = 20) had experienced an OC recurrence. Seven patients relapsed within 18 months and another seven within 36 months. A relapse within 24 months was described in a total of 10 patients.

**Table 1:** Patient and tumour characteristics, showing mean and standard deviation or frequency and percentage

		All Patients (*N* = 48)
Age (years)	Mean (SD)	58.7 (7.0)
	< 35	1 (2.0%)
	≥ 35, < 50	7 (14.3%)
	≥ 50, < 70	35 (71.4%)
	≥ 70	6 (12.3%)
*gBRCA* Mutation (n/%)	*BRCA1*	34 (69.3%)
	*BRCA2*	15 (30.7%)
Metachronous breast cancer (n/%)	Yes	15 (30.7%)
	Before HGSC	10
	Synchronous	1
	After HGSC	4
	No	34 (69.3%)
	No information available	0
Relapse (n/%)	Yes	20 (40.9%)
	No	29 (59.1%)
	No information available	0
Time to relapse (months)	≤ 18	7 (35%)
	> 18, ≤ 36	7 (35%)
	> 36, ≤ 60	6 (30%)
	> 60	0
	No information available	0
FIGO stage (n/%)	FIGO I	6 (12.3%)
	FIGO II	1 (2.0%)
	FIGO III	31 (63.3%)
	FIGO IV	9 (18.4%)
	No information available	2 (4.0%)
Histopathology (n/%)	Serous	45 (91.8%)
	No information available	4 (8.2%)
Operation (n/%)	Laparotomy	44 (89.8%)
	Laparoscopy	2 (4.0%)
	No information available	3 (6.2%)
Systemic treatment (n/%)	Neoadjuvant chemotherapy	8 (16.3%)
	Adjuvant chemotherapy	38 (77.5%)
	No information available	3 (6.1%)
PARPi (n/%)	Yes	26 (53.1%)
	No	20 (40.8%)
	No information available	3 (6.2%)

In > 80% of cases, the FIGO stage at initial diagnosis was stage III or higher. Only seven patients were diagnosed at FIGO stage I or II. In 45 cases (91.8%), the tumour was classified as serous carcinoma (high-grade: 44; low-grade: 1). A total of 44 patients underwent initial surgical treatment via laparotomy; only two patients underwent laparoscopy that was performed for diagnostic purposes prior to the initiation of neoadjuvant chemotherapy due to initially inoperable disease.

Neoadjuvant chemotherapy was administered in 16.3% of patients. Adjuvant systemic therapy was given to 77.5% (*n* = 38) of patients. In 28 cases, guideline-recommended first-line therapy with carboplatin and paclitaxel was administered. Moreover, 53.1% (*n* = 27) of patients received a PARP inhibitor (PARPi) as maintenance therapy.

### Management for high-risk patients

A total of 33 patients received a recommendation for IBCS, of whom 17 pursued the recommended surveillance (Table 2). Of those, 19 patients were advised to additionally undergo RRBM. 16 patients did not receive a recommendation for IBCS, and none of these patients were advised to undergo RRBM. Despite the absence of a formal recommendation, five of these 16 patients opted to participate in IBCS.

**Table 2:** Risk-reducing management (IBCS, RRBM/RRCM) in patients with *gBRCAmut* HGSC

		*n*/%
Intensified breast cancer surveillance (IBCS)	Recommendation IBCS	33 (67.3%)
	Additional recommendation RRBM	19
	No additional recommendation RRBM	14
	Attending IBCS programme	17
	Not attending IBCS programme	16
	No recommendation IBCS	16 (32.7%)
	Additional recommendation RRBM	0
	No additional recommendation RRBM	16
	Attending IBCS	5
	Not Attending IBCS	11
Risk-reducing bilateral mastectomy (RRBM)	Recommendation RRBM/RRCM	19 (38.8%)
	Additional recommendation IBCS	19
	No additional recommendation IBCS	0
	Receiving bilateral mastectomy	3
	Not receiving bilateral mastectomy	16
	No Recommendation RRBM/RRCM	30 (61.2%)
	Additional recommendation IBCS	14
	No additional recommendation IBCS	16
	Receiving bilateral mastectomy	2
	Not receiving bilateral mastectomy	27

All 19 patients for whom RRBM was recommended also received a recommendation for IBCS. Of these, three patients (15.8%) elected to undergo RRBM, and 16 patients (84.2%) declined the procedure despite the recommendation. Among the 30 patients who did not receive a recommendation for RRBM, two (6.8%) underwent the procedure, nonetheless. Of the patients who were not advised to undergo RRBM, 46.7% (*n* = 14) received a recommendation for IBCS.

### RRBM in patients with gBRCAmut HGSC

Five patients with *gBRCAmut* underwent RRBM following an initial diagnosis of HGSC (Table 3). All five patients had previously received a recommendation for IBCS, and three of them had participated in the surveillance programme. Three patients had also received a formal recommendation for RRBM (two of whom had additionally been advised to undergo IBCS).

**Table 3:** Characteristics and risk-reducing management in *gBRCAmut* HGSC patients undergoing bilateral mastectomy

bilateral mastectomy in patients with HGSC	Patient 1	Patient 2	Patient 3	Patient 4	Patient 5
Age at HGSC diagnosis (years)	52	55	56	53	33
FIGO	IIB	IB	IIIC	IA	IIIC
Recommendation IBCS	Yes	Yes	Yes	Yes	Yes
IBCS before surgery	No	Yes	Yes	No	Yes
Recommendation RRBM/RRCM	No	Yes	No	Yes	Yes
Age at breast surgery (years)	66	62	61	59	34
Time to operate after HGSC diagnosis (months)	168	86	64	66	18
Kind of mastectomy	SSM	SSM	MRM	MRM	SSM
Metachronous breast cancer	Yes	Yes	No	Yes	No
Relapse (months from first diagnosis onwards)	No	No	No	No	Yes (21)

Three of the five patients developed metachronous breast cancer, which was the primary reason to undergo mastectomy and risk reducing contralateral mastectomy (RRCM). The remaining two patients, who did not develop breast cancer, chose to undergo RRBM as a preventive measure.

In four patients, bilateral mastectomy was performed > 60 months after initial diagnosis and primary surgery. The fifth patient underwent RRBM 18 months after initial diagnosis. This patient was also the only one to experience a recurrence of cancer during the observation period, which occurred 21 months after diagnosis. None of the patients had been diagnosed with a HGSC-relapse prior to undergoing breast surgery.

Three patients received a skin-sparing mastectomy with implant-based reconstruction, and two patients opted for a modified radical mastectomy without reconstruction.

## Discussion

Forty-four of 49 patients with *gBRCAmut* HGSC did not undergo RRBM during follow-up. Only two patients underwent conventional RRBM as a preventive procedure. Three additional patients opted for mastectomy and RRCM as part of therapeutic surgery following an initial BC diagnosis. However, RRBM was recommended to a total of 19 patients, and IBCS was advised for a further 33 patients. Nevertheless, only about half of the patients ultimately pursued these measures.

The median age at diagnosis for OC in the general population is approximately 68 years (Schultz et al. 2025). In contrast, patients harbouring *gBRCA1/2* mutations typically develop the disease at a considerably younger age, with a reported median age of onset of 50–60 years (Kotsopoulos et al. 2018). This trend is consistent with the findings of the present cohort, in which 71.4% of patients were diagnosed between the ages 50 and 70 years, yielding a median age at diagnosis of 58.7 years. As the analysed cohort exclusively comprised patients with *gBRCAmut*, the earlier disease onset compared with the national median age of 68 years can be attributed to the genetic predisposition (Schultz et al. 2025).

HGSC is diagnosed at an advanced stage (FIGO stage III or higher) in > 70% of cases (Menon et al. 2021), a finding that was even more pronounced in our *gBRCAmut* cohort, where > 80% presented with FIGO stage III or IV disease. Consequently, after cervical cancer, HGSC remains the gynaecologic malignancy with the highest mortality rate worldwide. In approximately half of all patients, recurrence occurs rapidly, most commonly within the first 18–24 months following initial diagnosis (Perren et al. 2011, Burger et al. 2011, González-Martín et al. 2023, Caruso et al. 2025, Salas Bolívar et al. 2025). In our cohort, more than 40% of patients experienced a recurrence of HGSC, with 14 of 20 affected patients developing recurrent disease within the first three years.

A metachronous BC was identified in 15 patients (30.7%) in this analysis. In 10 patients (20.4%), however, BC was diagnosed before HGSC. In the general *gBRCAmut* HGSC population, the reported risk of developing BC within 5–10 years after the initial cancer diagnosis ranges between 6% and 11% (Evans et al. 2024, Domchek et al. 2013). Four patients in this analysis (8.3%) developed BC following the diagnosis of HGSC, which is consistent with the data reported in the literature. Recently published prospective data by Apostol et al. suggest that BC risk in *gBRCAmut* patients following a diagnosis of OC is likely substantially lower than previously assumed. Only 4.3% of patients developed BC after a median follow-up of 4.9 years (Apostol et al. 2026). The cumulative 15-year risk of BC was 11.5% and only three patients died as a direct consequence of BC (Apostol et al. 2026). However, the authors note that advances in targeted therapies for HGSC may significantly prolong survival in the future. A higher incidence of BC among *gBRCAmut* patients may therefore emerge in the coming years. PARPi are now established as part of standard adjuvant treatment (Ray-Coquard et al. 2019, Emons et al. 2025). The treatment was officially approved for clinical use in Germany in November 2020. In our cohort, more than half of the patients (53.1%) have already received maintenance therapy with a PARPi. More than 80% of patients were diagnosed with FIGO stage III or IV disease. Nevertheless, approximately one-third did not receive PARPi therapy. Consistent with the inclusion criteria of the PAOLA-1 Trial, PARPi treatment was offered only to patients with FIGO stage IIIB disease or higher (Ray-Coquard et al. 2019). As detailed tumor stage classifications were not available in the present analysis, further stratification was not feasible. In addition, it is likely that PARPi therapy was omitted in selected patients owing to individual clinical factors, such as comorbidities and impaired performance status.

To reduce cancer risk, patients with HBOC are advised to undergo risk-reducing surgeries, such as RRBM and RRSO. Alternatively, intensified surveillance programmes, such as IBCS of GCHBOC, may be used for early cancer detection (Bick 2021, Bick et al. 2019, Amann et al. 2023). In high-risk patients, IBCS involves annual breast MRI in combination with semi-annual breast ultrasound examinations. In cases of indeterminate findings, additional imaging in the form of mammography is performed. Two-thirds of our patients were recommended to undergo IBCS. The most common reason for not recommending IBCS was advanced age, as the programme ends at 70 years. A more cautious approach was also adopted in patients with advanced, inoperable OC and limited life expectancy.

The effectiveness of such surveillance programmes largely depends on patient adherence to scheduled examinations. For IBCS, adherence rates are assumed to be comparable with those of national BC screening programmes involving regular imaging, such as mammography, at approximately 75% (Kotsopoulos et al. 2018, Großmann et al. 2023, Heinig et al. 2023, Sedani et al. 2025, Gao et al. 2018, Lipscomb et al. 2020). In contrast, among patients with *gBRCAmut* HGSC, only 50% participated in the IBCS programme when it was recommended. The prolonged adjuvant systemic treatment and the high recurrence rate (40.9% of patients experienced relapse within the first five years; see Table 1) may contribute to the relatively low uptake of IBCS in this patient population.

During follow-up, four patients developed BC. However, in three cases, BC occurred more than five years after the initial OC diagnosis (see Table 3). Overall, fewer than 10% of patients were diagnosed with BC during the observation period. These real-world data are consistent with the recent findings reported by Apostol et al. (Apostol et al. 2026).

Although RRBM was recommended to 38.8% of patients, only 10% actually underwent the procedure (Table 2). Two of these patients opted for RRBM, without having received a formal medical recommendation to do so. Of the five patients receiving a bilateral mastectomy, only two patients underwent RRBM in a preventive setting. The three remaining patients opted for mastectomy of the affected breast plus RRCM after being diagnosed with BC. Consistent with previous findings, most patients diagnosed with HGSC did not choose RRBM. This may be attributed to the overall poor prognosis and the typically advanced HGSC tumour stage at diagnosis. To date, there are no established recommendations in the literature regarding the optimal timing of RRBM after an HGSC diagnosis. Recent high-quality prospective data suggest that a general recommendation for RRBM in gBRCAmut patients after HGSC should be omitted. Instead, RRBM should be discussed on an individual basis, not earlier than five years after the initial HGSC diagnosis (Apostol et al. 2026). Our real-world data support this proposed clinical approach. The majority of patients in this analysis underwent RRBM after more than five years of follow-up.

Considering the significantly increased risk of cancer recurrence within the first 18–36 months after HGSC diagnosis, the suggested time interval should be respected before undertaking RRBM (González-Martín et al. 2023). In addition to the disease-free interval, primary tumour stage and whether complete cytoreduction (no residual disease) was achieved, patient age should also be taken into account when making surgical decisions. A more conservative approach has previously been discussed on an individual basis for patients aged ≥ 60 years (Salyer et al. 2019, Metcalfe et al. 2019, Melanson et al. 2025). Ultimately, the decision for or against RRBM should be based on an individualised risk–benefit assessment. However, these real-world data show that most *gBRCA*mut HGSC patients opt against RRBM. These findings further support the conclusions of Apostol et al., suggesting that the risk of metachronous breast cancer in patients after *gBRCA*mut HGSC appears to be low.

Our study has several limitations. Data were gathered retrospectively and the study was conducted in a single-centre German setting, which is reflected in the limited sample size. In particular, no conclusions can be drawn for subgroups (for example patients with FIGO I or II HGSC). The recommendations are based on national clinical guidelines. Although these are broadly consistent with international guidelines, they may differ in certain aspects due to variations in socio-economic conditions and healthcare system structures. Further investigations, ideally through multicentre international collaborations, are warranted to provide stronger evidence and to better inform counselling and decision-making regarding preventive surgical strategies for *gBRCA*mut OC patients in remission.

### Conclusion

Our data confirms the results from recent prospective trials, showing that the risk of developing BC after HGSC appears to be considerably lower than previously assumed. The recommendation for RRBM appears to be appropriate only in selected clinical situations. HGSC patients themselves predominantly opt against surgical management, instead preferring enrolment in IBCS.

## Supplementary Information

Below is the link to the electronic supplementary material.

Supplementary Material 1

## Data Availability

No datasets were generated or analysed during the current study.

## Abbreviations

ACMG: American College of Medical Genetics and Genomics
BC: Breast cancer
BRCA: Breast cancer gene
gBRCAmut: Germline BRCA-mutated
BRCAwt: BRCAwild-type
DNA: Deoxyribonucleic acid
FIGO: Fédération Internationale de Gynécologie et d’Obstétrique
GCHBOC: German Consortium for Hereditary Breast and Ovarian Cancer (DK-FBREK)
HBOC: Hereditary breast and ovarian cancer
HGSC: High-grade serous carcinoma of the ovary, peritoneum or fallopian tube
IBCS: Intensified breast cancer surveillance
MRI: magnetic resonance imaging
MRM: Modified radical mastectomy
PARPi: Poly ADP ribose polymerase inhibitor
RRBM: Risk-reducing bilateral mastectomy
RRCM: Risk-reducing contralateral mastectomy
RRSO: Risk-reducing salpingo-oophorectomy
OC: Ovarian cancer
SSM: Skin-sparing mastectomy
