# Supplementary material for: Breast cancer prevention in patients with gBRCA-mutated ovarian cancer
Source: J Cancer Res Clin Oncol. 2026 Jun 21;152(6):124. doi: 10.1007/s00432-026-06543-4 (PMC13287192; doi:10.1007/s00432-026-06543-4)
Supplement: Supplementary file 1 — Supplementary Material 1 [file 432_2026_6543_MOESM1_ESM.docx]

| Patient | *BRCA* Mutation | Coding Change | Protein Change |
| --- | --- | --- | --- |
| 1 | BRCA1 | c.2293G> T | p.(Glu765Ter), heterozygot |
| 2 | BRCA1 | c.5406+4A>G heterozygot | No information |
| 3 | BRCA1 | c.181 T>G | p.(Cys61Gly), heterozygot |
| 4 | BRCA1 | c.213-12A>G | No information |
| 5 | BRCA1 | c.(? -200-1)_(441+1 442-l)del, heterozygot | No information |
| 6 | BRCA1 | c.181T>G | p.(Cys61Gly), heterozygot |
| 7 | BRCA1 | c.4986+5G>A | No information |
| 8 | BRCA1 | c.3403C>T | p.(Gln1135Ter), heterozygot |
| 9 | BRCA1 | c.5406+4A>G heterozygot | No information |
| 10 | BRCA1 | c.181T>G | p.(Cys61Gly), heterozygot |
| 11 | BRCA1 | c.1881_1884del heterozygot | p.(Ser628Glufs*3) |
| 12 | BRCA1 | c.1687C> T | p.(Gln563*), heterozygot |
| 13 | BRCA1 | c.1874_1877dup | p.(Val627SerfsTer4), heterozygot |
| 14 | BRCA1 | c.5492del | p.(Pro1831LeufsTer3), heterozygot |
| 15 | BRCA1 | c.5266dupC | p.(Gln1756Profs*74), heterozygot |
| 16 | BRCA1 | c.5266dupC | p.(Gln1756Profs*74) heterozygot |
| 17 | BRCA1 | c.(4986+1_ 4987-1)_(507 4+1_5075-1)del | p.(Asp1692Metfs*10), heterozygot |
| 18 | BRCA1 | c.5266dup | p.(Gln1756Profs*7 4), heterozygot |
| 19 | BRCA1 | c.3403C>T | p.(Gln1135*), heterozygot |
| 20 | BRCA1 | c.5266dupC | p.(Gln1756Profs*74), heterozygot |
| 21 | BRCA1 | c.3481_3491del11 | p.(Glu1161Phefs*3), heterozygot |
| 22 | BRCA1 | c.66dupA | p.(Glu23Argfs*18) heterozygot |
| 23 | BRCA1 | c.5492del | p.(Pro1831Leufs*3), heterozygot |
| 24 | BRCA1 | c.2477_2478delCA | p.(Thr826Argfs*4) heterozygot |
| 25 | BRCA1 | c.4222C>T | p.(Gln1408*), heterozygot |
| 26 | BRCA1 | c.3700_3704del5 | p.(Val1234Glnfs*8) heterozygot |
| 27 | BRCA1 | c.2411_2412del | p.(Gln804Leufs*5) heterozygot |
| 28 | BRCA1 | c.5096G>A | p.(Arg1699Gln) heterozygot |
| 29 | BRCA1 | c.5266dup | p.(Gln1756ProfsTer7 4), heterozygot |
| 30 | BRCA1 | c.1154G>A | p.(Trp385Ter) heterozygot |
| 31 | BRCA1 | g.41256139_ 41277260del | No information |
| 32 | BRCA1 | c.1016dup | p.(Val340GlyfsTer6), heterozygot |
| 33 | BRCA1 | c.3485del | p.(Asp1162ValfsTer48), heterozygot |
| 34 | BRCA1 | c.3018_3021del | p.(His1006Glnfs*17) heterozygot |
| 35 | BRCA2 | c.5386 5393del heterozygot | p.(Asp1796CysfsTer8) |
| 36 | BRCA2 | c.9371A>T | p.(Asn3124IIe), heterozygot |
| 37 | BRCA2 | c. 755 758del | p.(Asp252ValfsTer24), heterozygot |
| 38 | BRCA2 | c.5303_5304del | p.(Leu1768ArgfsTer5), heterozygot |
| 39 | BRCA2 | c.1813dup | p.(lle605AsnfsTer11), heterozygot |
| 40 | BRCA2 | c. 7878G>C | p.(Trp2626Cys) heterozygot |
| 41 | BRCA2 | c.7069_7070del | p.(Leu2357Valfs*2) heterozygot |
| 42 | BRCA2 | c.1309_1312delAAAG | p.(Lys43711efs*22), heterozygot |
| 43 | BRCA2 | c.3637G> T | p.(Glu1213*) heterozygot |
| 44 | BRCA2 | c.2251dup | p.(Thr751Asnfs*2), heterozygot |
| 45 | BRCA2 | c.7069_7070delCT | p.(Leu2357Valfs*2) heterozygot |
| 46 | BRCA2 | c.634_635delAG | p.(Arg212Lysfs*2), heterozygot |
| 47 | BRCA2 | c.3191C>G | p.(Ser1064Ter), heterozygot |
| 48 | BRCA2 | c.5303 5304del | p.(Leu1.768ArgfsTer5), heterozygot |
| 49 | BRCA2 | c.2283T>G | p.(Tyr761Ter), heterozygot |

**Supplement Table 1** Coding and protein change in g*BRCAmut* patients with HGSC.
